# RELATIONSHIP BETWEEN FIBER INTAKE AND CARDIOVASCULAR RISK FACTORS IN ADOLESCENTS WITH SYSTEMIC LUPUS ERYTHEMATOSUS

**DOI:** 10.1590/1984-0462/2021/39/2019316

**Published:** 2020-08-28

**Authors:** Marcella Lage Pinto Moreira, Flávio Sztajnbok, Denise Tavares Giannini

**Affiliations:** a Center for Adolescent Health Studies, Universidade do Estado do Rio de Janeiro, Rio de Janeiro, RJ, Brazil.

**Keywords:** Lupus erythematosus, systemic, Risk factors, Adolescent, Waist-height ratio, Dietary fiber, Lúpus eritematoso sistêmico, Fatores de risco, Adolescente, Razão cintura-estatura, Fibra alimentar

## Abstract

**Objective:**

To evaluate the fiber intake and the relationship with cardiovascular risk factors in adolescents with juvenile systemic lupus erythematosus.

**Methods:**

This is a cross-sectional in which adolescents with juvenile systemic lupus erythematosus were evaluated. The dietary consumption was assessed by the 24-hour recall; nutritional status was classified according to the Body Mass index/Age by Sex; abdominal obesity was assessed through waist circumference, waist-to-height ratio and glucose and lipid metabolism. The data were analyzed using Statistical Software for Professionals 14 and all statistical analyses used an alpha error of 5%.

**Results:**

52 patients were evaluated, with a mean age of 16.7±1.5 years. Inadequate fiber consumption occurred in 61.5% (n=32) of them. Average of waist circumference measures (81.4 vs. 75.5 cm; p=0.02), waist-to-height ratio (0.51 vs. 0.47; p=0.02) and systolic blood pressure (122.1 vs. 114.8 mmHg; p=0.03) were higher in those who had inadequate fiber intake. Among the cardiovascular risk factors evaluated, the waist/height ratio showed a significant negative correlation with fiber consumption (r=-0.3; p=0.04), that is, the higher the fiber consumption, the lower the value of the waist ratio /stature.

**Conclusions:**

Low dietary fiber intake in adolescents with systemic lupus erythematosus juvenile is related to higher abdominal adiposity and consequently with increased cardiovascular risk.

## RESUMO

**Objetivo:**

Avaliar o consumo de fibras e analisar a sua relação com os fatores de risco cardiovascular em adolescentes portadores de lúpus eritematoso sistêmico juvenil.

**Métodos:**

Trata-se de um estudo transversal em que foram avaliados adolescentes com lúpus eritematoso sistêmico juvenil. O consumo alimentar foi avaliado pelo recordatório de 24 horas; o estado nutricional, classificado de acordo com o índice de massa corporal/idade, segundo o sexo; e a obesidade abdominal, analisada por meio da circunferência da cintura e razão cintura/estatura e do metabolismo glicídico e lipídico. Os dados foram analisados no Statistical Software for Professionals 14, e todas as análises estatísticas usaram erro alfa de 5%.

**Resultados:**

Foram avaliados 52 pacientes, com média de idade de 16,7±1,5 anos. O consumo inadequado de fibras ocorreu em 61,5% (n=32) dos adolescentes e evidenciou que a média das medidas da circunferência da cintura (81,4 *vs*. 75,5 cm; p=0,02), a relação cintura/estatura (0,51 *vs*. 0,47; p=0,02) e a pressão arterial sistólica (122,1 *vs.* 114,8 mmHg; p=0,03) foram maiores naqueles que tiveram consumo inadequado de fibras. Entre os fatores de risco cardiovascular avaliados, a relação cintura/estatura apresentou correlação negativa significante com consumo de fibras (r=-0,3; p=0,04). Ou seja, quanto maior o consumo de fibras, menor o valor da relação cintura/estatura.

**Conclusões:**

A baixa ingestão de fibras da dieta nos adolescentes com lúpus eritematoso sistêmico juvenil está relacionada com maior adiposidade abdominal e, consequentemente, com maior risco cardiovascular.

## INTRODUCTION

Juvenile systemic lupus erythematosus (JSLE) is a chronic, multisystemic inflammatory disease. Its cause is unknown and it is autoimmune in nature, characterized by the presence of several autoantibodies.^1^ It evolves with periods of activity and remission, which can be triggered by genetic, infectious, hormonal, environmental and even psychological factors. It is considered to be an unpredictable disease, which affects several of the organism’s systems, which can often lead to vital organ failure or permanent compromise of its functions.^2^ Cardiovascular disease (CVD) represents the most important cause of morbidity and mortality, such as coronary heart disease, myocardial and pericardial diseases, heart failure, heart valve diseases and conduction disorders.

Autoimmunity, the inflammatory process of JSLE and the use of several drugs are directly related to changes in the lipid profile and the metabolism of lipoproteins in the disease, both in the active and remission phases. The pattern of dyslipidemia (DLP) in JSLE is characterized by high levels of triglycerides (TG) and very low density lipoproteins (VLDL), associated with lower levels of high density lipoprotein (HDL), which demonstrates that JSLE itself promotes conditions that are favorable to the atherogenic inflammatory process.^3^ ^,^ ^4^ Some classes of drugs, especially corticosteroids, can induce changes in nutritional status (NS) and obesity, systemic arterial hypertension (SAH), DLP, hyperinsulinemia and insulin resistance (IR).^5^

Nutritional assessment in adolescence is extremely important, as changes in body composition occur at this stage. Teenagers gain 25% of their final height and 50% of their body mass.^6^ Thus, nutritional assessment is especially in patients with JSLE, because of cardiovascular risk factors. For an effective nutritional assessment, it is necessary to verify the individual’s food consumption pattern. The use of the 24-hour food record (R24h) in studies has many advantages, as it is a fast, relatively inexpensive and easy-to-use instrument.^7^ The R24h is the method chosen by the European Food Consumption Survey Method (EFCOSUM Project).^8^

Adequate fiber intake in a normal diet seems to reduce the risk of developing some CVDs. Thus, it is important to emphasize that diet quality is linked to the restriction of sodium, cholesterol and saturated fat intake, in addition to adequate intake of fiber and other nutrients.^9^ The objectives of this study were to evaluate the food consumption of all nutrients and fibers and to analyze the relationship between fiber intake and cardiovascular risk factors in adolescents with JSLE in a center for adolescent health at a secondary care level.

## METHOD

This is a cross-sectional study conducted at the rheumatology outpatient service of the the Center for Adolescent Health Studies (*Núcleo de Estudos da Saúde do Adolescente* - NESA). The inclusion criteria included adolescents with JSLE between 12 and 18 years old, who signed the free and informed consent form. Pregnant patients and those who were unable to stand in an orthostatic or supine position for nutritional assessment were excluded.

The diagnosis was made by the physician, based on the diagnostic criteria, according to the Systemic Lupus International Collaborating Clinics (SLICC),^10^ when there were at least four of the 17 criteria, at least one clinical criterion and one laboratory criterion. The Systemic Lupus Erythematosus Disease Activity Index (SLEDAI)^11^ was used to assess disease activity in the last 10 days. A cut-off point ≥3 was adopted to classify the disease activity. As for the use of medications such as corticosteroids and antimalarials, the data was dichotomized into use and non-use, and as a cutoff point, continuous administration of at least one month of use was instituted.

NS was assessed using objective methods, such as anthropometric, laboratory and dietary data. Weight was measured in kg, with an electronic scale (Micheliti^®^, São Paulo, Brazil), with a precision of 0.1 kg and a maximum capacity of 200 kg. For height (cm), a stadiometer fixed to the wall (Sanny^®^, São Paulo, Brazil) was used, with an accuracy of 0.1 cm. The subject was standing, barefoot, with their body in an anatomical position and head parallel to the ground, according to the Frankfurt plan.^12^ Weight and height measurements were used to assess the adolescent’s NS by calculating the body mass index (BMI) for age and sex. The classification was in accordance with the World Health Organization (WHO)^13^ for children and adolescents from 5 to 19 years of age in the following categories:

- Z score <-2: thinness.
- Z score between ≥-2 and <+1: normal weight.
- Z score between ≥ +1 and <2: overweight.
- score Z≥ +2: obesity.

Waist circumference (WC) measurements were performed using an inelastic anthropometric tape, with a 0.1 cm scale. WC was measured at the midpoint between the lower border of the last rib and the upper border of the iliac crest at the end of a normal exhale, as proposed by the WHO.^13^ For classification, the graphs of risk of developing metabolic complications were used, according to sex.^14^ The waist/height ratio (WHR) was calculated by dividing WC (cm) by height (cm), and values ≥0.5 were indicative of high WHR.^15^ Arterial blood pressure (BP) was measured in the arm using an aneroid sphygmomanometer and cuff (Missouri^®^, São Paulo, Brazil), which fit the teenager’s circumference when he or she was sitting, relaxed, resting for 3- 5 minutes, in a calm environment.^16^ BP was considered altered if values ≥ 90th percentile, according to the classification of The Fourth Report on the Diagnosis, Evaluation, and Treatment of High Blood Pressure in Children and Adolescents, since this classification already shows lifestyle changes.^17^

For the metabolic evaluation, the following variables were taken into account: TG, total cholesterol (TC), HDL, low density lipoprotein (LDL) and fasting glycemia (FG). Blood collection was performed by venipuncture in a specialized laboratory, and it was preceded by a 12-hour fast. The levels of TG, TC and HDL were measured by the enzymatic colorimetric method and LDL was calculated using the Friedewald formula.^18^ The results were interpreted using the parameters of the I Atherosclerosis Prevention Guideline in Childhood and Adolescence,^19^ considering a change in lipid profile TC values to be ≥170, LDL ≥130, HDL <45 and TG ≥130 mg/dL or using a lipid-lowering agent. The serum glucose concentration was determined using an enzymatic method with hexokinase, and values above 100 mg/dL were considered to be elevated.^14^

The evaluation of food consumption was carried out using the R24h on a typical day. The following were questioned: the amount consumed, the size of the portions, the utensils used, and the way the food was prepared. Quantitative analysis of the diet was performed using the Avanutri^®^ Revolution 4.0 software (Rio de Janeiro, Brazil). The diet data analyzed were: calorie (Kcal), carbohydrate (CHO), protein (PTN), lipid (LIP), saturated fat (SF), monounsaturated fat (MF), polyunsaturated fat (PF), cholesterol (COL), calcium (Ca), zinc (Zn), selenium (Se), sodium (Na), vitamin D (vit. D) and fibers. According to Dietary Reference Intakes (DRI),^20^ the following nutritional recommendations were considered to be adequate:

- CHO: 50-60% of the total energy value (TEV).
- PTN: around 15% of the TEV.
- LIP: 25-35% of the TEV, with <7% of the TEV of SF, up to 20% of MF, up to 10% of PF and <200 mg/day of COL, up to 2000 mg of Na/day, 1,300 mg of Ca/day, 5 mcg of vit. D/day, 40 to 55g of Se/day for both sexes and 8-9 g of Zn/day for girls and 8-11 g of Zn/day for boys.

The total fiber for girls between nine and 18 years old is 26 g/day; and for boys between 9 and 13 years old, it is 31 g/day, and between 14 and 18 years old it is 38 g/day.

The data were stored in a spreadsheet of Excel version 7 softward and analyzed in Statistical Software for Professionals (STATA Version 14, São Paulo, Brazil). The distribution of variables as to normality was assessed using the Kolmogorov-Smirnov test. Continuous variables were presented as mean and standard deviation, and categorical variables were presented as absolute frequency and relative frequency. Comparisons between continuous variables were performed using the Student’s *t* test or the Mann-Whitney U test and a one-way analysis of variance, or the Kruskal-Wallis test. For comparisons of categorical variables, the χ^2^ test or Fisher’s exact test were applied. The correlation between variables of interest was performed using the Pearson or Spearman correlation coefficient. All of the statistical analyzes used an alpha error of 5%.

The study was approved by the Research Ethics Committee (*Comitê de Ética em Pesquisa* - CEP) of the Hospital Universitário Pedro Ernesto (HUPE), CEP/HUPE registration: 064245/2014 and Certificate of Presentation for Ethical Appreciation (CAAE): 34223714.6.0000.5259. The adolescents and their guardians signed the free and informed consent forms.

## RESULTS

The study sample consisted of 52 adolescents with JSLE. Four (8%) patients were male and 48 (92%) were female, with a mean age of 16.7±1.5 years (female: 16.6±1.5 years *vs*. male: 16.9 ± 1.6 years; p=0.39). The mean time of diagnosis of JSLE was 4.0±2.8 years, and the disease activity index was 4.3±5.6. Of the patients, 50% had SLEDAI >3 at the time of the consultation. The comorbidities found were SAH (48%; n=25), DLP (44.2%; n=23), obesity (13.5%; n=7) and hyperglycemia (9.6%; n=5). According to medication usage, 57.7% (n = 30) of the patients evaluated used corticosteroids, with an average dosage of 29.0±22.1 mg/day. Other drugs indicated were antimalarials (78.8%; n=41), Ca and vit. D supplements (53.8%; n=28), in addition to antihypertensive drugs (36.5%; n=19).

There was no significant difference between the sexes regarding anthropometric variables:

- BMI: female: 23.1 ± 4.2 kg/m^2^ *vs*. male: 22.5 ± 8.3kg/m^2^ (p =0.39);
- WC: female: 79.1±9.9 cm *vs*. male: 79±18 cm (p = 0.49);
- WHR: female: 0.49 ± 0.06 *vs*. male: 0.47 ± 0.1 (p = 0.21).

Regarding the classification of NS according to the BMI, a low frequency of thinness (9.6%; n=5) and a high frequency of excess weight (38.5%; n=20) were observed. Regarding the accumulation of abdominal visceral fat, 30.8% (n=17) of the patients had elevated WC and 42.3% (n = 22) had high WHR. The anthropometric and clinical-laboratory evaluations of the patients are in Table 1. There were no differences in lipid and glycid metabolism or BP among the different nutritional classifications.

**Table 1:** Mean and standard deviation of anthropometric measurements and clinical and laboratory characteristics according to nutritional diagnosis of adolescents with juvenile systemic lupus erythematosus.

	Low weight (n = 5)	Normal weight (n = 27)	Overweight (n = 13)	Obese (n = 7)	Total (n = 52)	p-value
Weight (kg)	42.9±5.5	54.3±7.4	65.6±8.0	80.5±10.5	59.6±13.0	<0.001
Height (m)	1.58±0.07	1.60±0.05	1.61±0.07	1.60±0.07	1.60±0.06	0.890
BMI (kg/m^2^)	16.9±0.8	21.1±2.3	25.2±1.4	31.3±2.1	23.1±4.5	0.490
WC (cm)	68.6±5.8	74.1±6.7	84.8±5.4	95.3±9.5	79.1±10.5	<0.001
WHR (cm)	0.43±0.04	0.46±0.04	0.52±0.03	0.60±0.05	0.49±0.06	<0.001
SBP (mmHg)	112.0±19.2	117.6±20.2	119.8±10.4	127.1±7.5	119.0±16.7	0.440
DBP (mmHg)	72.0±13.0	74.8±14.1	74.4±10.4	80.0±11.5	75.2±12.6	0.714
FG (mg/dL)	81.0±17.2	82.8±11.1	84.5±10.1	88.7±8.9	83.9±11.1	0.603
TC (mg/dL)	183.2±44.5	179.4±69.6	165.9±43.7	170.8±36.3	174.8±55.6	0.896
HDL-c (mg/dL)	63.6±15.1	59.2±36.3	51.1±13.6	46.4±9.0	55.4±26.7	0.584
LDL-c (mg/dL)	99.4±27.5	105.2±54.4	94.3±34.0	107.2±29.2	101.9±43.1	0.890
TG (mg/dL)	101.8±45.5	119.0±89.6	104.5±77.1	98.4±19.2	110.1±74.3	0.897

BMI: body mass index; WC: waist circumference; WHR: waist/height ratio; SBP: systolic blood pressure; DBP: diastolic blood pressure; FG: fasting blood glucose; TC: total cholesterol; HDL: high density lipoprotein; LDL: low density lipoprotein; TG: triglycerides.

The R24h showed an average consumption of 2,124.5±830.0 Kcal/day, 265.5±120.7g of CHO/day, 91.3±43.3 g of PTN/day and 67.3 ± 39.1 g of LIP/day. The average distribution of macronutrients in the diet was adequate for CHO (51.9%) and LIP (29.9%) and high for PTN (18.2%). The consumption characteristics of macro and micronutrients, according to NS, are described in Table 2. Approximately 35% (n=17) of the evaluated patients had high consumption of COL and 50% (n = 26) of SF. Ca was the only micronutrient with a statistical difference between the sexes: 383.2 mg (±347.0) for girls *vs*. 619.3 mg (±169.6) for boys (p=0.05).

**Table 2:** Total energy consumption, macro and micronutrients and fibers in adolescents with juvenile systemic lupus erythematosus according to nutritional status.

	Low weight (n = 5)	Normal weight (n = 27)	Overweight (n = 13)	Obese (n = 7)	Total (n = 52)	p-value
TEV (kcal)	2031±629	2031±870	2325±715	2209±1061	2125±830	0.747
CHO (g)	272±95	253±117	270±115	302±170	266±121	0.904
CHO (%)	53.1±4.4	51.4±9.7	51.3±10.3	54.0±7.4	51.9±9.0	0.899
PTN (g)	76.6±19.7	97.2±50.5	89.3±37.7	82.1±35.2	91.3±42.3	0.835
PTN (%)	16.3±5.9	19.8±7.4	17.3±4.9	15.4±5.0	18.3±6.5	0.385
LIP (g)	52.9±11.5	65.0±43.3	74.1±39.3	74.6±36.8	67.3±39.1	0.665
LIP (%)	31.6±3.6	28.9±6.9	31.3±11.2	30.5±5.2	29.9±7.6	0.757
COL (mg)	281±311	202±147	223±118	119±72	204±156	0.161
SF (g)	16.4±7.3	15.7±13.7	27.5±21.3	13.5±7.1	18.2±15.4	0.283
PF (g)	14.0±11.3	8.8±6.3	16.6±8.3	9.7±7.3	10.6±7.6	0.312
MF (g)	18.0±10.8	14.1±11.9	21.5±17.7	12.7±8.7	16.0±13.1	0.678
Fiber (g)	15.5±5.1	20.9±9.9	18.2±9.2	18±9.9	19.3±9.3	0.640
Na (mg)	2721±988	2308±1373	2931±1390	2609±1955	2537±1418	0.420
Zn (mg)	9.1±3.7	8.6±7.0	8.6±6.0	8.0±7.5	8.5±6.5	0.879
Se (mg)	63.5±52.4	46.3±35.0	56.8±35.6	32.6±41.5	48.6±37.7	0.251
Ca (mg)	341±160	380±301	445±292	453±628	402±342	0.697
Vit. D (mcg)	0.8±0.6	1.1±1.1	1.5±1.1	0.4±0.5	1.1±1.0	0.115

TEV: calories; CHO: carbohydrate; PTN: protein; LIP: lipid; COL: cholesterol; SF: saturated fat; PF: polyunsaturated fat; MF: monounsaturated fat; Na: sodium; Zn: zinc; Se: selenium; Ca: calcium; vit. D (vitamin D).

In this study, high Na intake was observed, regardless of NS, and all of the subjects showed inadequate intake of Ca and vit. D. The average consumption of Se and Zn was also lower than DRI in most patients - 67.3% (n = 35) and 57.7% (n = 30), respectively.

Inadequate fiber intake occurred in 61.5% (n=20) of the adolescents, with an average intake of 19.3±9.3 g/day (Table 2). The mean of the measures was higher in those who had inadequate fiber intake (Table 3):

- WC: 81.4 *vs*.75.5 cm; p=0.02;
- WHR: 0.51 *vs*. 0.47; p=0.02;
- SBP: 122.1 *vs*. 114.8 mmHg; p=0.03.

**Table 3:** Mean and standard deviation of anthropometric and biochemical measurements according to the total fiber consumption of adolescents with juvenile systemic lupus erythematosus.

	Adequate intake (n = 20)	Inadequate intake (n = 32)	Total (n = 52)	p-value
Age (years)	16.4±1.4	17.0±1.5	16.7±1.5	0.070
Weight (kg)	56.3±11.0	61.7±14.1	59.6±13	0.070
Height (m)	1.6±0.1	1.6±0.0	1.6±0.1	0.490
BMI (kg/m^2^)	21.8±4.0	23.9±4.7	23.1±4.5	0.050
WC (cm)	75.5±9.5	81.4±10.8	79.1±10.5	0.020
WHR	0.47±0.06	0.51±0.06	0.49±0.06	0.020
SBP (mmHg)	115±15	122±18	119±17	0.030
DBP (mmHg)	71.2±11.1	78.1±13.1	75.2±12.6	0.060
FG (mg/dL)	84.8±12.5	83.1±10.2	83.9±11.1	0.310
TC (mg/dL)	162.1±46.8	181.9±60.7	174.8±55.6	0.120
HDL-c (mg/dL)	47.2±12.2	59.4±31.7	55.4±26.7	0.060
LDL-c (mg/dL)	91.1±29.2	108.6±49.2	101.9±43.1	0.090
TG (mg/dL)	124.6±82.1	102.5±70.7	110.1±74.3	0.170

BMI: body mass index; WC: waist circumference; WHR: waist/height ratio; SBP: systolic blood pressure; DBP: diastolic blood pressure; FG: fasting blood glucose; TC: total cholesterol; HDL: high density lipoprotein; LDL: low density lipoprotein; TG: triglycerides.

Regarding the relationship between fiber intake and CV risk factors, a negative correlation was observed between fiber intake and WHR (r=-0.3; p=0.04), that is, the higher the fiber intake, the lower the WHR value, as described in Table 4.

**Table 4:** The correlation between total fiber intake of adolescents with juvenile systemic lupus erythematosus and anthropometric and laboratorial measures.

	r	p-value
Weight (kg)	-0.2	0.220
BMI (kg/m^2^)	-0.2	0.130
WC (cm)	-0.2	0.080
WHR	-0.3	0.040
SBP (mmHg)	-0.1	0.520
DBP (mmHg	-0.2	0.180
FG (mg/dL)	0.2	0.190
TC (mg/dL)	-0.1	0.540
HDL-c (mg/dL)	-0.2	0.130
LDL-c (mg/dL)	-0.1	0.520
TG (mg/dL)	0.1	0.340

BMI: body mass index; WC: waist circumference; WHR: waist/height ratio; SBP: systolic blood pressure; DBP: diastolic blood pressure; FG: fasting blood glucose; TC: total cholesterol; HDL: high density lipoprotein; LDL: low density lipoprotein; TG: triglycerides.

## DISCUSSION

Our study found that a minority of patients had adequate fiber intake, which can be harmful, since fibers reduce glycemia and lipid absorption and contribute to the prevention and treatment of obesity.^21^ It is common for teenagers to replace their main meals with fast food and high calorie density foods, skipping some meals throughout the day and consuming a low fiber diet, due to the low intake of fruits and vegetables.^22^

International studies with adolescents show the association between low fiber intake and excess weight.^23^ This worrisome relationship was also found here, since excess adiposity is related to IR, which directly triggers the metabolic syndrome in these patients.^24^ Additionally, in this study, it was observed that the average of WHR was significantly lower in those with adequate fiber intake. Furthermore, the study showed a negative correlation between WHR and fiber consumption. Corroborating these results, studies have proven the effectiveness of the use of the WHR as a more accurate indicator of visceral adipose tissue and for having a strong correlation with CVD. The WHR incorporates WC as a measure of abdominal adiposity, but is adjusted to the individual’s size by dividing it by height. It exhibits a good predictive value and association with other cardiovascular risk conditions, such as DLP and combined risk factors, which reinforces the recommendation to screen adolescents at risk.^25^

The main comorbidities found were SAH and DLP. Epidemiological studies maintain that fibers protect against CVD. In fact, 10 cohort studies in the United States and Europe, with a 6-10-year follow-up, concluded that fiber was associated with a 14% reduction in the risk of coronary events and a 27% risk of coronary death.^26^ These results can be explained by the effect of fibers on blood pressure and C-reactive protein (CRP) levels, as their intake was inversely associated with CRP in the National Health and Nutrition Examination Survey1999-2000.^26^

The mean of the SBP measurements in the present study was significantly higher in those who had inadequate fiber intake. Probable mechanisms to explain this situation include the fact that fibers act to improve hyperinsulinemia, IR and to reduce body weight.^27^ King et al.^27^ noted that supplementation with soluble fiber for 12 weeks was able to reduce SBP and DBP.

In addition, dietary intervention to control hypercholesterolemia must emphasize the importance of consuming foods rich in fiber, especially soluble foods (found in oats, fruits and legumes). Insoluble fibers, on the other hand, contribute to reducing weight or abdominal circumference, since they induce greater satiety through their intrinsic physical properties.^28^ Studies show that fiber intake is inversely associated with plasma levels of homocysteine and inflammatory markers such as interleukin-6 and CRP.^27^ ^,^ ^28^ The relationship between the intake of vitamin B6 and B12, folate and dietary fiber with disease activity was also investigated. Hyperhomocysteinemia may be related to atherosclerosis, inflammation and the activation of these markers in autoimmune diseases.^24^

In the present study, it was noted that 50% of patients consumed excess SF, and a diet rich in SF contributes to the maintenance of DLP in the disease.^29^ In addition, high protein intake was seen in the diet, regardless of NS. The study by Caetano et al.,^30^ carried out with 22 children and adolescents with lupus, revealed that excessive protein consumption determines constant bone mineral loss in these patients, and it is known that these individuals already have a high risk of developing low bone mineral density.

Osteoporosis related to the use of corticosteroids results from a negative Ca balance. Corticosteroids decrease the absorption of Ca in the gastrointestinal tract and increase its elimination through the urinary tract.^30^ This fact is aggravated in our patients, as they had low consumption of Ca (approximately 1/3 of the DRI for girls and 1/2 for boys) and of vit. D (about 1/5 of the DRI for both sexes) and were using a moderate number of corticosteroids (30 mg/day).^31^

A higher prevalence of vit D. deficiency has been demonstrated in JSLE patients compared to healthy individuals or those with other rheumatological diseases.^31^ The photosensitivity characteristic of the disease and the recommendation regarding the use of sunscreen determine the individual’s less exposure to the sun, decreasing the cutaneous production of vit. D. Regular use of corticosteroids and antimalarials appears to alter the metabolism of this vitamin.^31^ The American College of Rheumatology (ACR) recommends guidelines to reduce bone loss in patients with JSLE treated with corticosteroids. Lifestyle changes and a Ca-rich diet are also suggested.^32^ Ca (> 1500 mg) and vit. D (20 µg or 800 IU) supplementation is indicated in cases where food intake is difficult.^32^ In this study, most patients used these supplements.

All patients had a high consumption of Na. Adequate Na consumption should not exceed 3 g/day for patients with lupus nephritis or SAH, whether or not it is secondary to corticotherapy.^33^ Low consumption of Se and Zn, important nutrients in the immune response, was also found. Zn deficiency promotes an immune dysfunction that mainly affects Th cells (involved in cellular immunity), which can cause sensorineural disorders and reduced body mass. Se increases anti-inflammatory properties, and has an important effect on the maturation of T cells, and the response of autoantibodies of the dependent T cell.^33^

The article, despite having a small sample, is original and made up of a population of adolescents with a rare disease. It evaluated information about the NS and the dietary profile that are related to CVDs, such as R24h, abdominal obesity and glucose and lipid metabolism. The results point to the importance of a balanced and fiber-rich diet in the metabolic and, consequently, inflammatory control of the disease, in the maintenance of NS, and in the reduction of cardiovascular risk, which is very common in lupus patients. It would be interesting to extend the study, and promote a dietary intervention between the two groups in order to assess laboratory differences in blood tests. This intervention could contribute positively to those who did not adhere to the improvement of eating patterns, as a motivational factor for changing eating habits.

In conclusion, fiber intake appears to be associated with a significant reduction in WHR, suggesting the possibility of a reduced risk of CVD in patients who consume more fiber. Thus, it is extremely important to assess and monitor the NS of patients with JSLE and create strategies that encourage the intake of adequate fiber and other nutrients.
